# Peroxisome proliferator-activated receptors (PPARs)-independent functions of fish oil on glucose and lipid metabolism in diet-induced obese mice

**DOI:** 10.1186/1476-511X-9-101

**Published:** 2010-09-16

**Authors:** Masaki Wakutsu, Nobuyo Tsunoda, Sachiko Shiba, Etsuko Muraki, Keizo Kasono

**Affiliations:** 1 Department of Clinical Dietetics and Human Nutrition, Faculty of Pharmaceutical Sciences, Josai University, 1-1 Keyakidai, Sakado, Saitama 350-0295, Japan

## Abstract

**Background:**

Fish oil is known to improve lifestyle-related diseases. These effects occur partly via activation of PPARs by the n-3 polyunsaturated fatty acids included abundantly in fish oil. We investigated fish oil functions on glucose and lipid metabolism that are both dependent on and independent of PPARs pathway.

**Methods:**

Mice were fed a diet containing 30 en% beef tallow (B diet) for twelve weeks to induce obesity. The mice were then divided into two groups which were fed either a B diet or a diet containing 30 en% fish oil (F diet). Each group was further divided into two groups which were administered PPARα and γ antagonists or vehicle once a day for three weeks.

**Results:**

The F diet groups showed lower triglyceride levels in plasma and liver than the B diet groups, but PPARs antagonists did not affect the triglyceride levels in either diet groups. The F diet groups also showed improvement of glucose tolerance compared with the B diet groups. However, PPARs antagonists made glucose tolerance worse in the F diet group but improved it in the B diet group. Therefore, by the administration of antagonists, glucose tolerance was inversely regulated between the B and F diets, and hypolipidemic action in the plasma and liver of the F diet group was not affected.

**Conclusion:**

These results suggest that fish oil decreases lipid levels in plasma and liver via PPARs pathway-independent mechanism, and that glucose tolerance is inversely regulated by PPARs antagonists under diets containing different oils.

## Background

It is known that fish oil improves lifestyle-related diseases like diabetes and hyperlipidemia [1,2]. It has been reported that one of the main mechanisms of the effects is the activation of peroxisome proliferator-activated receptors (PPARs) by n-3 polyunsaturated fatty acids found abundantly in fish oil [3]. PPARs are a family of transcriptional factors that exist in three isoforms: PPARα, PPARγ, and PPARδ [4]. PPARα is abundantly expressed in liver and is associated with the lipid- and lipoprotein-lowering properties by up-regulation of fatty acid oxidation, while PPARγ is predominantly expressed in adipose tissue and generally affects adipocyte differentiation and adipose tissue lipid distribution by induction of adipogenesis to recruit new small adipocytes [5]. PPAR δ is ubiquitously expressed and affects lipid metabolism and insulin sensitivity [4,6]. However, functional relationship between fish oil and PPAR δ has not been well understood.

Fibrates and thiazolidinediones (TZDs) are well known drugs activating PPARα and PPARγ, respectively. Fibrates activate PPARα and decrease hepatic triglyceride production by increasing fatty acid oxidation in hyperlipidemic patients [7,8]. TZDs activate PPARγ and increase insulin sensitivity in diabetic patients [9-11].

Although these drugs are composed of simple chemical components, fish oil contains many types of fatty acids and unknown components. Therefore, fish oil also exerts its functions through mechanisms that are independent of PPARs. For example, the activity of a number of lipogenic enzymes such as fatty acid synthase (FAS) and stearoyl-CoA desaturase are conspicuously decreased by fish oil. It has been reported that these enzymes expression levels are tightly controlled by sterol regulatory element binding protein 1 (SREBP1) transcriptional factor [12,13]. Although there has been several reports concerning the relationship between PPARs and fish oil in the glucose and lipid metabolism [14-17], it is not well understood how PPARs participate in fish oil functions.

In this study, we investigated a specific and PPARs-independent pathway of fish oil functions in diet-induced obese mice using a PPARα and γ antagonists mixture.

## Methods

### Animals

Female ddY mice were obtained from Saitama Experimental Animals Supply Co. Japan (Tokyo, Japan) at 5 weeks of age. They had free access to a standard diet pellet (MF; Oriental Yeast, Tokyo, Japan) and water for 1 week to accommodate to the new environment before the experiments began. The mice were maintained at a constant temperature of 23 ± 3°C and humidity of 55 ± 10% with a fixed artificial light cycle (12 hour light/dark cycle). All procedures were approved by the Josai University Animal Care and Use Committee and complied with the National Institutes of Health's Guide for the Care and Use of Laboratory Animals.

### Diets

Experimental diets were consisted of 30% fat and 25% sucrose on a calorie basis. The composition of the diets was based on the AIN-93G [18] with modifications as described previously [19]. Beef tallow or fish oil was used in the diets instead of soybean oil (B diet or F diet, Table 1).

**Table 1:** Experimental compositions^1^.

	B diet	F diet
	g/100 g diet	
Fish oil	-	13.0
Beef tallow	13.0	-
Casein	21.5	21.5
Sucrose	26.4	26.4
Corn starch	28.6	28.6
Vitamin mix 2	1.1	1.1
Mineral mix 3	3.8	3.8
Cellurose	5.4	5.4
L-cystine	0.3	0.3
T-butylhydroquinone	0.003	0.003
		
Energy, kcal/100 g	408.0	404.4
Fat energy ratio, %	29.9	29.3
Sucrose energy ratio, %	25.0	25.2

^1 ^Based on the AIN-93G (30) and modified.

^2, 3 ^Vitamin and mineral mix were based on the AIN-93G formation.

Casein, sucrose, β-starch, vitamin mixture, mineral mixture, cellulose powder and beef tallow were purchased from Oriental Yeast (Tokyo, Japan). L-cystine and t-butylhydroquinone were purchased from Wako Pure Chemical Industries, Ltd. (Osaka, Japan), and fish oil was a gift from the NOF CORPORATION (Tokyo, Japan). The diets were made following a previous procedure [19].

### Experimental procedures

The mice were given free access to the MF diet or each experimental diet and water for all periods. The control group was fed the MF diet for all periods. Other mice were fed the B diet for twelve weeks to induce obesity, and then divided into two groups which were fed either the B diet or F diet. Each group was further divided into two groups which were injected with PPARα and γ antagonists mixture or vehicle (B(+), B(-), F(+) and F(-) groups) once a day for three weeks. One mg/kg body weight of the PPARα antagonist MK886 (Wako Pure Chemical Industries, Ltd., Osaka, JAPAN) and 3 mg/kg body weight of the PPARγ antagonist bisphenol-A-diglycidyl ether (BADGE; Tocris Cookson Inc., Missouri, USA) were subcutaneously injected.

MK886 acts as an inhibitor of PPARα through blockage of the conformational change which is necessary for PPARα activation [20,21]. BADGE is a competitive inhibitor of ligand of PPARγ [16,22]. We ascertained that MK886 (1 mg/kg body weight) and BADGE (3 mg/kg body weight) inhibited 60-70% of the expression of acyl-CoA oxidase (ACO) gene in liver and CD36 gene in WAT, which were up-regulated by PPARα or γ respectively, at 24 h after the single injection of the mixture of both inhibitors in ddY mice fed the fish oil diet. The efficacy of each inhibitor in the mixture was the same as when individually administered (data not shown).

The body weight of the mice was measured once weekly during the experimental period. The energy intake was measured at 13 wk. An oral glucose tolerance test (OGTT) was conducted at 14 wk under a 6-h fasting condition. The mice were killed at 15 wk after 4-h fast with an intraperitoneal injection of Somnopentyl (Kyoritsu Seiyaku Corporation, Tokyo, Japan). Blood samples were collected from the postcaval vein, and the plasma was separated by centrifugation (9100 × g for 10 minutes at 4°C). It was stored at -30°C for analyses. Weight of the liver and parametrial white adipose tissue (WAT) were measured. Then, the liver was immediately frozen in liquid nitrogen, and stored at -80°C for analyses and measurement of lipids and mRNA. Parametrial white adipose tissue was fixed in freshly prepared 10% (w/v) buffered formalin for measurement of the adipocyte surface area.

### Measurement of food intake

At 13 wk, food intake and fecal weight were measured [23]. Food intake and fecal weight are shown as the average for 3 days. The standard error of food intake was calculated from the variation of daily intake of each group.

### Oral glucose tolerance test (OGTT)

At 14 wk, D-glucose (1 mg/g body weight; Glucose Injection, DAIICHI SANKYO COMPANY, LTD., Tokyo, Japan) was orally administered by a stomach tube after 6-h fast. Blood samples were obtained by cutting the tail end before and 15, 30, 60 and 120 minutes after glucose administration. Blood glucose levels were measured using the glucose analyzer ASCENSIA DEXTER ZII (Bayer Health Care, Osaka, Japan). Plasma insulin levels were measured using an insulin ELISA kit (Morinaga Institute of Biological Science Inc., Yokohama, Japan).

### Measurement of lipid levels in plasma and liver

The TG and TC levels in plasma were measured by colorimetric slides using the FUJI DRICHEM analyzer (DRI-CHEM 3500, FujiFilm Medical Co. Ltd., Tokyo, Japan).

The liver sections (100-200 mg) were homogenized in phosphate buffer solution (pH7.4) by a polytron (PT 3100, KINEMATICA, Inc., Littau/Lucerne, Switzerland), and crude lipid extracts were obtained by the method described by Bligh and Dyer [24]. TG levels were measured using crude lipid samples by the Wako TG E test kit (Wako Pure Chemical Industries, Ltd., Osaka, Japan).

### RNA isolation and measurement of mRNA levels by real-time RT-PCR

Total RNA was extracted from the liver using TRIzol Reagent (Invitrogen, USA). Quantitative real-time PCR analysis was performed on 0.5 μg of total RNA with an iCycler iQ (Bio-Rad Laboratory, Inc., Tokyo, Japan) and the ABI Prism 7000 thermal cycler (Applied Biosystems, Tokyo, Japan) using QuantiTect SYBR Green RT-PCR (QIAGEN, Germany) with gene-specific primers. The mRNA levels of liver in all groups were represented as ratios to the mRNA levels in the B (-) group.

### Measurement of adipocyte sectional area

Adipose tissue was fixed in freshly prepared 10% (w/v) buffered formalin, embedded in paraffin, and the sections were stained with hematoxylin-eosin. Three different representative microscopic fields were captured manually from sections of each animal and quantitated with an image analysis system (Image J, Wayne Rasband, NIH). The mean adipocyte area was calculated from more than 600 cells per mouse in each group.

### Statistical analysis

Statistical comparisons of the groups were made by one-way ANOVA, and each group was compared with the others by Fisher's PLSD test (Protected least significant difference test) (Statview 5.0; SAS Institute Inc., USA). *P *values less than 0.05 were considered to indicate statistical significance. Values are means ± SE.

## Results

### Energy intake, fecal weight, final body weight and organs weight

In the B (+) group, energy intake was significantly increased and fecal weight was also increased compared with other groups (Table 2). Final body weight and organs weight were not different in all groups (Table 2).

**Table 2:** Energy intake, fecal weight, final body weight and organs weight.

	MF	B(-)	B(+)	F(-)	F(+)
Energy intake (kcal)	11.23 ± 0.87^a^	10.23 ± 3.27^a^	12.63 ± 1.21^b^	10.73 ± 2.06^a^	10.04 ± 1.66^a^
Fecal weight (g)	2.81 ± 0.17^a^	1.01 ± 0.12^bc^	1.14 ± 0.14^b^	0.99 ± 0.05^bc^	0.76 ± 0.04^c^
					
Final body weight (g)	33.8 ± 2.7	39.2 ± 2.4	38.4 ± 3.9	40.0 ± 2.2	40.5 ± 2.0
Liver (g)	1.37 ± 0.10	1.51 ± 0.11	1.67 ± 0.16	1.50 ± 0.06	1.58 ± 0.21
WAT (g)	1.34 ± 0.50	2.41 ± 0.44	2.68 ± 0.93	2.56 ± 0.29	2.82 ± 0.86
Gastrocnemius muscle (g)	0.33 ± 0.02	0.26 ± 0.03	0.31 ± 0.02	0.30 ± 0.02	0.32 ± 0.02

B(-): B diet, B(+): B diet + antagonists mixture, F(-): F diet, F(+): F diet + antagonists mixture. Food intake is shown as the average for 3 days. The standard error of food intake was calculated from the variation of daily intake of each group. Data is shown as mean ± SE (n = 3 - 4). Significance of difference is indicated as different alphabet: *p *< 0.05.

### Effects of fish oil on lipid metabolism under the administration of PPARs antagonists

Plasma total cholesterol levels and liver triglyceride accumulation significantly decreased in the F(-) group compared with the B(-) group (Table 3). Plasma triglyceride levels and total lipid accumulation in the liver showed a tendency to decrease in the F(-) group compared with the B(-) group. In the presence of PPARs antagonists, the liver lipid levels slightly increased in the B(+) group compared with the B(-) group, but did not change in the F(+) group compared with the F(-) group in all parameters.

**Table 3:** Lipids levels in plasma and liver at 15 wk

	MF	B(-)	B(+)	F(-)	F(+)
Plasma					
TG (mmol/L)	1.16 ± 0.17^a^	0.80 ± 0.15^ab^	0.94 ± 0.13^a^	0.55 ± 0.04^b^	0.49 ± 0.05^b^
TC (mmol/L)	1.62 ± 0.32^ab^	2.14 ± 0.31^b^	2.37 ± 0.20^b^	1.34 ± 0.15^a^	1.58 ± 0.30^ab^
Liver					
Total lipid (mg/g liver)	42.8 ± 5.5^ac^	101.2 ± 19.5^ab^	129.9 ± 40.6^b^	52.3 ± 9.0^ac^	35.2 ± 5.5^c^
TG (mg/g liver)	9.9 ± 1.3^a^	44.0 ± 9.3^b^	44.7 ± 8.3^b^	12.6 ± 1.5^a^	8.9 ± 1.9^a^

B(-): B diet, B(+): B diet + antagonists mixture, F(-): F diet, F(+): F diet + antagonists mixture. Data is shown as mean ± SE (n = 3 - 4). Significance of difference is indicated as different alphabet: *p *< 0.05.

**Figure 1:**
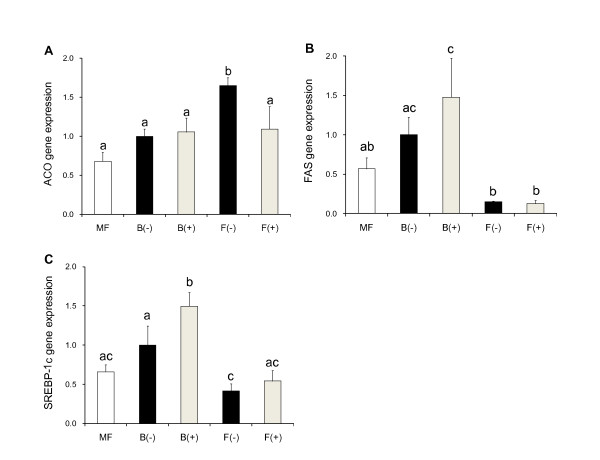
**Expression of genes in liver**. Mice were killed at 15 wk after 4 h fasting. Liver was removed and gene expression levels of acyl-CoA oxidase (ACO), fatty acid synthase (FAS) and sterol regulatory element binding protein-1c (SREBP-1c) (A, B, C) were measured by real time RT-PCR. Gene expression levels are indicated as relative levels to those of the B(-) group. Data is shown as mean ± SE (n = 3 - 4). Significance of difference is indicated as different alphabet: *p *< 0.05.

ACO gene expression in the liver significantly increased in the F(-) group compared with the B(-) group (Figure 1A). It was significantly suppressed by PPARs antagonists in the F(+) group (Figure 1A). While, FAS and SREBP1-c genes expression in the liver remarkably decreased in the F(-) group compared with the B(-) group, but there was no change between the F(-) and F(+) groups (Figure 1B, C). These mRNA levels slightly increased in the B(+) group compared with the B(-) group. FAS and SREBP1-c genes expression levels were compatible with the plasma and liver lipid levels (Figure 1B, C, Table 3).

### OGTT

**Figure 2:**
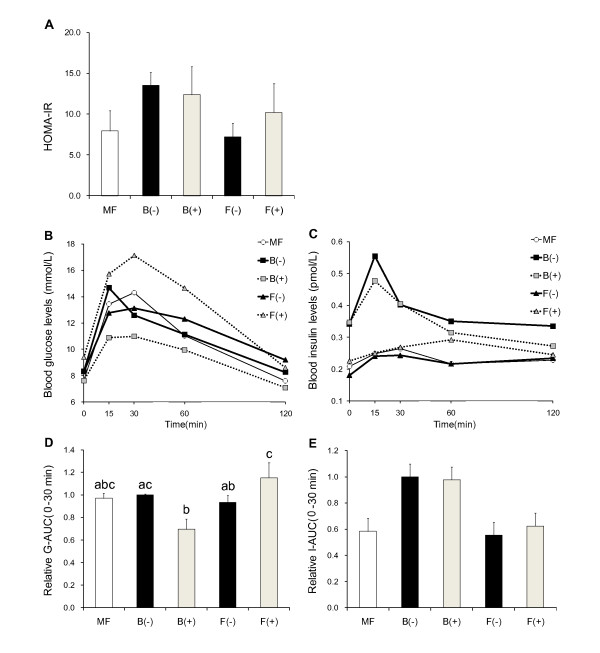
**Oral glucose tolerance tests (OGTT)**. After the injection of PPARs inhibitor for two weeks, a glucose solution was administered by intragastric gavage at a dose of 1 g/kg body weight after 6 h fasting. HOMA-IR calculated by the blood glucose levels and the plasma insulin levels at 0 min (A). The blood glucose and plasma insulin concentration were measured at 0, 15, 30, 60 and 120 min after glucose administration (B, C). Glucose area under the curve (G-AUC) and insulin area under the curve (I-AUC) calculated over 30-min periods after oral glucose administration (D, E). G-AUC and I-AUC are represented as the relative levels to those of the B(-) group. Data is shown as mean ± SE (n = 3 - 4). Significance of difference is indicated as different alphabet: *p *< 0.05.

To investigate the effect of fish oil under the administration of PPARs antagonists on glucose metabolism, we performed OGTT after 2 weeks administration of PPARs antagonists. HOMA-IR calculated by the blood glucose levels and the plasma insulin levels at 0 min in OGTT was decreased by half in the F(-) group compared with the B(-) group, although it was not significantly different in both B and F diet groups in the presence of PPARs antagonists (Figure 2A).

Compared with the B(-) group, the blood glucose levels at 15 min in OGTT decreased in the F(-) group (Figure 2B). In the presence of PPARs antagonists, the blood glucose levels of the B(+) group decreased at all indicated times compared with the B(-) group, while the blood glucose levels of the F(+) group remarkably increased at 0, 15, 30 and 60 min compared with the F(-) group (Figure 2B). Similarly, in the blood glucose area under the curves (AUC) between 0 and 30 min, the B(+) group was significantly decreased compared with the B(-) group, whereas the F(+) group was significantly increased compared with the F(-) group (Figure 2D).

The plasma insulin levels at all times in OGTT and the plasma insulin AUC were decreased in the F(-) group compared with the B(-) group (Figure 2C, E). In the presence of PPARs antagonists, there was no effect on the plasma insulin levels and plasma insulin AUC in both diet groups (Figure 2C, E).

### Adipocyte size

**Figure 3:**
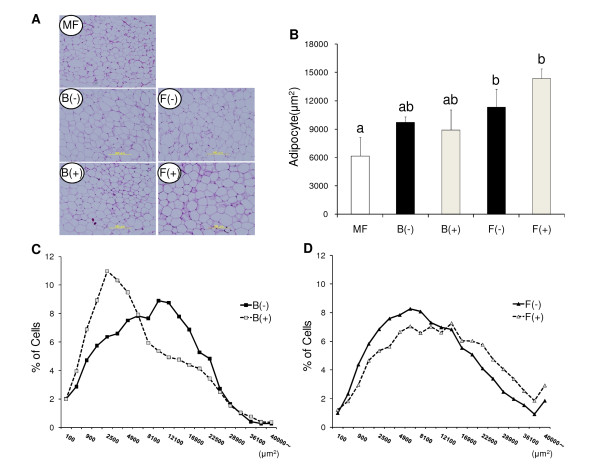
**Representative histological section of white adipose tissue (WAT)**. Representative histological section of white adipose tissue stained with hematoxilin-eosin. A) Histology. B) Mean adipocyte area is given as μm^2^. Data is shown as mean ± SE (n = 3 - 4). Significance of difference is indicated as different alphabet: *p *< 0.05. C) D) The data are expressed as the percentage of cells found in a given adipocyte area.

There was no significant difference in the mean sizes of the adipocytes amomg 4 groups (B(-), B(+), F(-), F(+)) (Figure 3A, B). The distribution of the sizes of the adipocytes is shown in Figure 3C and 3D. The peak of distribution in B(-) group was about 10,000 μm^2 ^and that in F(-) group was about 5,000 μm^2^. The distribution of adipocyte sizes in the B(+) group was smaller than that in the B(-) group. However, the distribution of adipocyte sizes in the F(+) group was larger than that in the F(-) group.

## Discussion

We have investigated fish oil functions independent of the PPARα and γ pathway in diet-induced obese mice using a PPARα and γ antagonists mixture. By the administration of the antagonists, the hypolipidemic action in the plasma and liver was not affected. However, glucose tolerance was inversely regulated by the antagonists between the B diet and F diet. This study suggests that PPARs play more important roles in glucose metabolism than lipid metabolism in diet-induced obese mice treated with a fish oil diet.

Although many previous studies have reported that fish oil activated PPARs [25-27], it is not well understood how PPARs participate in fish oil functions. The previous studies have supported the presence of PPARα-independent pathway in lipid metabolism in PPARα-defeicent mice or fish oil diet fed mice [14,15]. In our study, triglyceride levels in the plasma and liver of the F diet groups were decreased compared with the B diet groups, but PPARs antagonists did not affect triglyceride levels in both B and F diet groups (Table 3). Thus, it suggests that the main mechanisms of fish oil functions on lipid metabolism are PPARs-independent in diet-induced obese mice. In the liver of the F diet groups, ACO gene expression levels were decreased, and FAS and SREBP-1c genes expression levels were not changed in the presence of PPARs antagonists (Figure 1). Therefore, these results indicate that suppression of fatty acid synthesis is more important than induction of fatty acid oxidation by fish oil in liver. These observations indicate that fish oil functions on lipid metabolism are mainly controlled by SREBP-1c, which regulates fatty acid synthesis, and the functions are independent of PPARs. It is reported that a heterodimer with retinoid X receptor (RXR) and liver X receptor (LXR) activates the expression of SREBP-1c, whereas a heterodimer with RXR and PPARα promotes ACO gene expression [28,29]. Then, it is thought that PPARα suppression by the antagonist activates SREBP-1c pathway through induction of RXR/LXR formation. These cross-talks are possible to induce up-regulation of SREBP-1c and FAS genes and slight increase of lipid levels in liver in B diet groups in this study (Table 3 and Figure 1). On the other hand, it is thought that the F diet directly suppressed fatty acid synthesis by down-regulation of SREBP1-c and FAS genes. Then our study supports the importance of PPARα-independent pathway on lipid metabolism under the F diet.

In contrast, improvement of glucose metabolism by fish oil was prevented by PPARs antagonists (Figure 2). This result suggests that PPARs play more important roles in glucose metabolism in the F diet groups. Although Neschen et al have examined the association of PPARα or γ to plasma adiponectin levels and insulin sensitivity in fish oil-fed wild type or PPARα null mice [16,17], there has been few reports concerning the functional relationship between PPARs and fish oil in glucose metabolism. By the administration of PPARs antagonists, blood glucose levels in OGTT increased in the F diet group, but decreased in the B diet group. Several reports observed that inhibition of PPARs also affected plasma insulin levels [30,31]. However, in our study, PPARs antagonists did not change insulin levels in OGTT in the F and B diets (Figure 2). These results find that the roles of PPARs on glucose metabolism are influenced by dietary oils.

To elucidate the cause of the inverse regulation of insulin sensitivity by PPARs antagonists in the B and F diet groups, we observed the sizes of adipocyte. It has been reported that small adipocytes improved insulin sensitivity [32-34] and that a diet containing fish oil decreased the size of adipocyte [35]. In the distribution of the adipocytes, the adipocytes in the F(-) group were smaller than the B(-) group. In the presence of PPARs antagonists, the distribution of the adipocytes was shifted to smaller in the B groups and larger in the F groups (Figure 3). It has been reported that internal PPARγ mediates adipocyte hypertrophy and insulin resistance under long term high fat (beef tallow) diet [36]. Therfore, PPARγ antagonist induced small adipocytes by suppression of lipid accumulation inducing adipocyte hypertrophy, and enhanced insulin sensitivity in the B diet group. On the other hand, administration of high amount of PPARγ agonist also induced adipogenesis recruiting small adipocytes from preadipocytes [37]. Therefore, PPARγ antagonist inhibited the function of fish oil as a strong PPARγ stimulator, and increased large adipocytes and induced insulin resistance.

Our results suggest that there is a PPARs-independent pathway of fish oil functions on lipid metabolism, and that PPARs play a more important role in glucose metabolism. SREBP-1c inhibited by fish oil is the main mechanism of lipid level reduction in mice fed fish oil. It is also possible that PPARs-stimulating drugs as well as PPARs inhibitors show different or inverse effects under diets containing different oils.

## Competing interests

The authors declare that they have no competing interests.

## Authors' contributions

MW carried out the animal care, all experiments and analysis, and conceived of the study and its design, and wrote the manuscript. NT participated in the animal care and OGTT, and conceived of the study, and participated in its design and coordination and helped to draft the manuscript. SS participated in the animal care and the measurement of food intake. EM participated in the histological analysis of adipocyte. KK conceived of the study, and participated in its design and coordination and helped to draft the manuscript. All authors read and approved the final manuscript.
